# Transcranial Magnetic Stimulation in Alzheimer’s Disease: Are We Ready?

**DOI:** 10.1523/ENEURO.0235-19.2019

**Published:** 2020-01-06

**Authors:** Marina Weiler, Kevin C. Stieger, Jeffrey M. Long, Peter R. Rapp

**Affiliations:** Neurocognitive Aging Section, Laboratory of Behavioral Neuroscience, National Institute on Aging, Intramural Research Program, Baltimore, Maryland 21224

**Keywords:** brain stimulation, excitatory/inhibitory balance, therapeutic development

## Abstract

Transcranial magnetic stimulation (TMS) is among a growing family of noninvasive brain stimulation techniques being developed to treat multiple neurocognitive disorders, including Alzheimer’s disease (AD). Although small clinical trials in AD have reported positive effects on cognitive outcome measures, significant knowledge gaps remain, and little attention has been directed at examining the potential influence of TMS on AD pathogenesis. Our review briefly outlines some of the proposed neurobiological mechanisms of TMS benefits in AD, with particular emphasis on the modulatory effects on excitatory/inhibitory balance. On the basis of converging evidence from multiple fields, we caution that TMS therapeutic protocols established in young adults may have unexpected detrimental effects in older individuals or in the brain compromised by AD pathology. Our review surveys clinical studies of TMS in AD alongside basic research as a guide for moving this important area of work forward toward effective treatment development.

## Abstract (precis)

Transcranial magnetic stimulation (TMS) is among a growing family of noninvasive brain stimulation techniques being developed to treat multiple neurocognitive disorders, including Alzheimer’s disease (AD). Although small clinical trials in AD have reported positive effects on cognitive outcome measures, significant knowledge gaps remain, and little attention has been directed at examining the potential influence of TMS on AD pathogenesis.

## Significance Statement

There is an urgent need for the development of new, effective strategies in the battle against Alzheimer’s disease (AD). Transcranial magnetic stimulation (TMS) has emerged as a promising possibility, but evidence regarding long-term efficacy and mechanism of action is limited. Among the major unresolved issues, findings linking the effects of TMS on excitatory/inhibitory balance with mechanisms of AD pathogenesis merit careful consideration. Our survey of clinical TMS studies in AD alongside basic research aims to move the area forward toward effective treatment development using noninvasive brain stimulation.

## The need for novel approaches to AD treatment

Alzheimer’s disease (AD), the most common form of dementia, is characterized by progressive memory impairment and associated decline in multiple cognitive domains, ultimately leaving patients incapacitated. Inexorably eroding the lifetime of memories that defines us, AD robs patients of their unique identity. The neuropathological hallmarks of AD prominently include microscopic foci of degenerating neurites and extracellular amyloid β-protein (Aβ) deposition, together with intracellular aggregates of hyperphosphorylated tau protein that disrupt microtubule organization (Selkoe, 2001). The single greatest risk for AD is aging. The ε4 allele of the apolipoprotein E (APOE) gene is present in ∼40% of cases and is the strongest genetic risk for the sporadic, late-onset form of AD (Farrer et al., 1997; Heffernan et al., 2016). In the absence of effective interventions for disease prevention or slowing, the projected burden of AD represents a looming health-care crisis as the population of most industrialized countries continues to grow older.

Currently approved pharmacological treatments for AD offer limited symptomatic relief for some patients, and none alter the underlying progression of disease. While the search for new drugs with improved clinical efficacy is ongoing, increasing attention is focused on disease-modifying strategies aimed at bending the trajectory of aging toward healthy neurocognitive outcomes. Ideally, intervention would be initiated in at-risk individuals before the clinical expression of disease, during the decades-long prodromal phase thought to precede AD diagnosis. Noninvasive brain stimulation (NIBS) has generated considerable interest in this context. Prominently including transcranial magnetic stimulation (TMS) and transcranial direct current stimulation, this family of related technologies shares a generally well tolerated safety profile in healthy young adults and is currently under investigation for treating a growing list of potential indications (Rossi et al., 2009; Guo et al., 2017).

Among the various types of NIBS, TMS has received the greatest attention in clinical research on neuropsychiatric disorders. The mechanistic basis of TMS benefits is poorly understood, but there is general agreement that cortical excitability can be persistently modified by the repetitive delivery of a high-intensity magnetic field, generated by passing electrical current through an inductive coil. Repetitive TMS (rTMS), delivered in daily hour-long sessions over the course of several weeks was approved by the US Food and Drug Administration (FDA) for the treatment of pharmacologically refractory depression in 2008. In general, trials of rTMS treatment versus sham showed significant improvement in depression scores and lower rates of remission with rTMS (Health Quality Ontario, 2016; Pohar and Farrah, 2019), benefits that can be enhanced when rTMS is combined with antidepressant medication (Wei et al., 2017). In the ensuing years, the range of potential clinical applications under investigation has increased dramatically, including a number of relatively small trials in AD (Table 1).

**Table 1:** Summary characteristics of clinical studies using rTMS to treat AD

Authors	Sample	Exclusion/ inclusion criteria	Methods	Stimulation site	Cognitive outcome variable	Assessment schedule	Summary results	Author conclusions
Cotelli et al., 2006	15 mild to moderate AD patients	Exclusion of patients with major depression	One session of 20 Hz rTMS during cognitive stimulation. No sham group	Unilateral dlPFC and sham region	Action naming and Object naming	Baseline and during stimulation	Patients improved action naming accuracy during stimulation with rTMS applied to either the right or left dlPFC.	High-frequency TMS could represent a potential treatment for language deficits in AD patients.
Cotelli et al., 2008	12 mild AD, 12 moderate to severe AD patients	Exclusion of patients with major depression	One session of 20 Hz rTMS during cognitive stimulus. No sham group	Unilateral dlPFC and sham region	Action naming and Object naming	Baseline and during stimulation	Mild AD improved action naming accuracy during stimulation with rTMS applied to either the right or left DLPFC. Moderate to severe AD improved action and object naming accuracy with rTMS applied to either the right or left DLPFC.	High-frequency TMS could represent a potential treatment for language deficits not only in the early phase of AD, but also in more advanced stages.
Cotelli et al., 2011	10 moderate AD patients	Exclusion of patients with major depression	Two groups: a 4 week stimulation group, and 2 week placebo treatment + 2 weeks of stimulation. 20 Hz rTMS, for 25 min/d, 5 d/week. No sham group	dlPFC (hemisphere not specified)	MMSE, ADL, IADL, Picture naming, SC-BADA, Aachen Aphasia Test, serial curve position, Cognitive estimation test	Baseline, 2, 4, and 12 weeks after stimulation onset	The 4 week stimulation group improved on SC-BADA after the first 2 weeks of stimulation. The placebo + real stimulation group only improved on SC-BADA after the 2 weeks of stimulation. Effects lasted for 8 weeks in both groups.	High-frequency TMS has long lasting effects on auditory sentence comprehension performance in moderate AD patients.
Drumond Marra et al., 2015	34 MCI subjects	Exclusion of patients with psychiatric disorders	Sham and stimulation groups. 10 Hz for 5 s, 25 s intertrain interval 20 min/d for 5 d/week for 2 weeks	Left dlPFC	IQCODE, B-ADL, MMSE, RBMT, Logical memory I and II, RAVLT, Letter-number sequencing test, Digit span, TMT A/B, Verbal fluency tests, Victoria Stroop Test	Baseline, end of treatment and 30 days after end of treatment	MCI improved RBMT scores after 10 Hz stimulation, lasting up for 30 d. MCI improved TMT-B 30 d after treatment. Sham improved Logical memory, letter-number sequencing and TMT-B after treatment. Effects on the Logical memory lasted up for 30 d. Sham improved verbal fluency 30 d after treatment.	High-frequency rTMS may represent an effective intervention for MCI and could delay further decline.
Bentwich et al., 2011	7 mild or moderate AD patients	Inclusion of 2 patients with depression and 4 patients with depression in remission	No sham groups. rTMS-COG. Intensive + maintenance phase (4.5 months of stimulation total). 10 Hz for 2s, 20 trains	Broca, right/left dlPFC, Wernicke, right/left pSAC	ADAS-cog, CGIC, MMSE, ADAS-ADL, HAMILTON, NPI	Baseline, after intensive phase, and after maintenance phase	Improved ADAS-cog scores after 6 weeks and 4.5 months of treatment. No significant changes on other tests.	High-frequency TMS combined with cognitive training may have a synergistic effect and improve cognition for up to 4.5 months.
Ahmed et al., 2012	32 mild to moderate AD, 13 severe AD patients	N/A	Sham, 20 Hz and 1 Hz groups. 20 Hz: 5s, 20 trains. 1 Hz: 2 trains of 1000 s, 30 s intertrain interval. 5 d	Bilateral dlPFC	MMSE, IADL, GDS	Baseline, end of treatment, 1 and 3 months after treatment	Mild to moderate AD improved in all tests after 20 Hz up to 3 months compared to 1 Hz and sham. Mild to moderate AD improved in IADL after 1 Hz compared to sham. There was no improvement in severe AD.	High-frequency TMS has long lasting effects in mild to moderate AD and is more effective than low-frequency stimulation.

Turriziani et al., 2012	100 healthy control subjects, 8 MCI subjects	Exclusion of MCI subjects with history of psychiatric disorders	Sham and stimulation groups. One session of 1 Hz and iTBS applied in controls, 1 Hz applied in MCI. iTBS: 20 trains, three 50 Hz pulses (a burst) repeated at 5 Hz for 2s. 1 Hz: 600 pulses	Unilateral dlPFC for healthy controls and bilateral dlPFC for MCI (interval of 3 weeks)	Recognition memory for faces, buildings and words.	Immediately after stimulation	Recognition memory improved in controls and MCI after 1 Hz stimulation over the right dlPFC. iTBS over right dlPFC impaired nonverbal recognition memory in healthy controls. iTBS over left dlPFC had no effect in healthy controls.	Low frequency TMS over the right dlPFC improves recognition memory when applied during encoding in MCI and healthy controls.
Rabey et al., 2013	15 mild to moderate AD patients	N/A	Sham and stimulation groups. rTMS-COG. Intensive phase + maintenance phase (4.5 months in total). 10 Hz, 20 trains, for 2 s.	Broca, right/left dlPFC, Wernicke, right/left pSAC	ADAS-cog, CGIC, NPI	Baseline, after intensive phase and after maintenance phase	AD patients improved on ADAS-cog and CGIC scores at the end of intensive phase. Effects lasted up for 4.5 months.	rTMS-COG treatment significantly improves cognition, is superior to currently available medications, and better than COG or TMS alone.
Eliasova et al., 2014	3 MCI and 7 mild AD patients	N/A	Sham-controlled study with a crossover design. 2 sessions of 10 Hz, 45 trains of 4.9 second duration with an interval of 25 s, resulting in 2250 pulses/session. One-day interval between each session	Right inferior frontal gyrus and right superior temporal gyrus (active rTMS), and vertex (sham rTMS)	TMT, Stroop test, complex visual scene encoding task test	Baseline and immediately after each stimulation	Stimulation over the inferior frontal gyrus induced significant improvement in the TMT A and B. No significant difference in the Stroop test or in the CVSET after the rTMS of the right inferior frontal gyrus.	Modulating the inferior frontal gyrus excitability with rTMS may lead to clinically relevant improvement in attentional task performance in early AD patients.
Rutherford et al., 2015	Stage 1: 10 mild to moderate AD patients; Stage 2: 6 mild to moderate AD patients	Exclusion of patients with moderate or severe depression. Inclusion of one patient with mild depression	4 week block of double-blind treatment with sham condition (Stage 1) followed by 2 weeks of open-label maintenance treatment repeated every 3 months (Stage 2). 20 Hz (40 pulses per burst) with 5-second inter-train intervals during cognitive task. 2000 pulses to each side	Both the left and right DLPFC per session	ADAS-cog, RMBC, MoCA	Stage 1: baseline and 4 weeks after the treatment. Stage 2: a few days after the treatment. MoCA was assessed every week in both stages	Stage 1: no statistically significant changes on ADAS-cog or RMBC scores comparing treated vs sham. Treated patients scored higher on MoCA in 2 and 3 weeks after start of treatment compared to baseline. Stage 2: with the exception of the ADAS-cog scores for 2 patients, all decline rates were better than the expected.	rTMS can be an effective tool for improving the cognitive abilities of patients with early to moderate stages of AD. However, the positive effects of rTMS may persist for only up to a few weeks. Specific skills being practiced during rTMS treatment may retain their improvement for longer periods.
Rabey and Dobronevsky, 2016	30 mild to moderate AD patients	N/A	No sham groups. rTMS-COG. Intensive phase only (6 weeks). 10 Hz, 20 trains for 2 s	Broca, right/left dlPFC, Wernicke, right/left pSAC	ADAS-cog, MMSE	Baseline and end of treatment	AD patients improved on ADAS-cog and MMSE scores at the end of treatment.	Repeated rTMS-COG treatment might be used to improve patients' cognitive status and maintain improvement over time.

Lee et al., 2016	19 mild AD, 7 moderate AD patients	Exclusion of patients who had taken psychoactive medications within a month of the study	Sham and stimulation groups. rTMS-COG. Intensive phase (6 weeks). 10 Hz, 20 trains for 2 s	Broca, right/left dlPFC, Wernicke, right/left pSAC	ADAS-cog, CGIC, MMSE, GDS	Baseline, end of treatment and 6 weeks after end of treatment	Mild AD patients improved in ADAS-cog after treatment and remained for 6 weeks, but no different than the sham group. The mild AD group also improved in MMSE 6 weeks after end of treatment. Sham group improved in GDS scores at the end of the treatment.	rTMS-COG is a useful adjuvant therapy with currently available medication for AD, especially during the mild stage of the disease.
Nguyen et al., 2017	2 MCI, 1 mild AD, and 4 moderate-to-severe AD patients	N/A	No sham group. rTMS-COG. Intensive phase + maintenance phase (4.5 months in total). 10 Hz, 20 trains, for 2 s	Broca, right/left dlPFC, Wernicke, right/left pSAC	ADAS-cog, MMSE, Dubois score, Frontal Assessment battery, Stroop color test, locomotor score, apathy score, caregiver burden interview and dependence score	Baseline, after intensive phase and 6 months after end of treatment	Patients improved on ADAS-cog, locomotor, apathy and dependence scores after intensive phase. Scores returned to baseline 6 months after treatment.	AD patients can benefit from rTMS-COG in terms of cognitive performance, apathy and independence. The duration of the benefit suggests that the repetition of a full course of stimulation every 6 months might be sufficient to produce a sustained clinical effect.
Zhao et al., 2017	30 mild to moderate AD patients	Exclusion of patients with a history of alcohol abuse or who had taken psychoactive medications within the past month	Sham and stimulation groups. 20 Hz, 20 s intermediate/train. 1 session/day, 5 d/week for 6 weeks	Parietal P3/P4 and posterior temporal T5/T6 according to electroence-phalogram system	ADAS-cog, MMSE, MoCA, WHO-UCLA AVLT	Baseline, end of treatment and 6 weeks after the end of treatment	Patients improved on ADAS-cog, MMSE, MoCA and WHO-UCLA AVLT after the treatment. 6 weeks following treatment, patients further improved on ADAS-cog and WHO-UCLA AVLT remained higher. The sham group also improved on ADAS-cog compared to pretreatment.	rTMS improves cognitive level, memory and language of AD patients, especially in the mild stage. Thus, rTMS can be recommended as a promising adjuvant therapy combined with cholinesterase inhibitors at the mild stage of AD patients.
Koch et al., 2018	14 mild AD	AD confirmed by CSF protein levels	Sham and stimulation groups (crossover design). Two weeks of 20 Hz stimulation (40 trains, for 2 s, 1600 pulses/d)	Precuneus	RAVLT, DSST, MMSE and FAB	Baseline and end of treatment	Patients improved on the Delayed Recall of RAVLT at the end of treatment. No significant effects after sham stimulation.	High-frequency rTMS is a promising treatment for memory impairment in patients at early stages of AD.

ADAS-ADL, Alzheimer Disease Assessment Scale-Activities of Daily Living subscale; B-ADL, Bayer Activities of Daily Living Scale; DSST, Digit Symbol Substitution Test; FAB, Frontal Assessment Battery; HAMILTON, Hamilton Depression Scale; IQCODE, Informant Questionnaire on Cognitive Decline in the Elderly; MoCA, Montreal Cognitive Assessment; NPI, Neuropsychiatric Inventory; pSAC, parietal somatosensory association cortex; RAVLT, Rey Auditory Verbal Learning Test; RMBC, Revised Memory and Behavior Checklist; RBMT, Rivermead Behavioral Memory Test; SC-BADA, Battery for Analysis of Aphasic Deficits; TMT A/B, Trail Making test A and B; WHO-UCLA AVLT, World Health Organization University of California-Los Angeles, Auditory Verbal Learning Test.

## Therapeutic effects of rTMS in AD

Developing NIBS as a potential intervention for any clinical indication critically involves the choice of an appropriate stimulation protocol. Generally, rTMS protocols are operationally classified as “low frequency” or “high frequency,” and “conventional” or “patterned.” Low-frequency typically refers to stimulation rates ≤1 Hz, whereas rates ≥3 Hz are considered high frequency (including the 10 and 20 Hz frequencies most commonly used in AD trials). In conventional protocols, single TMS pulses are applied in a regular rhythm; in patterned rTMS, short, high-frequency bursts are interleaved with brief periods of no stimulation. Some examples of patterned rTMS include stimulation mimicking theta activity, wherein short bursts of high-frequency pulses repeated at 5 Hz [theta burst stimulation (TBS)] are delivered as continuous TBS or intermittent TBS (iTBS) pulses. Perhaps most important with respect to the clinical effects of stimulation, low-frequency rTMS protocols are understood to result in cortical suppression and inhibition, whereas high-frequency stimulation increases cortical facilitation and excitability (Huang et al., 2005). Beyond stimulation frequency, a wide variety of generally untested factors are likely to influence the outcome of rTMS, including coil shape, coil–cortex distance, motor threshold normalization, area of stimulation, use of concomitant medication, and machine output, among others (Lang et al., 2006; Kar, 2019).

Initial studies of rTMS effects in AD focused on high-frequency protocols almost exclusively (Table 1, procedural details of available rTMS trials). For example, in research examining language function, mild and moderate AD participants received 20 Hz unilateral TMS over the dorsolateral prefrontal cortex (dlPFC; Cotelli et al., 2006, 2008). Object naming ability improved during stimulation, and the endurance of these effects, immediately after and 8 weeks following treatment, was assessed in subsequent work (Cotelli et al., 2011). The duration of intervention was also manipulated, with one group receiving a 4 week course of rTMS, while a second underwent 2 weeks of sham treatment followed by 2 weeks of rTMS. Auditory sentence comprehension improved in both groups, and although the previously reported effect on naming was not confirmed, comprehension benefits persisted for 8 weeks. Other outcome measures were unaffected, including activities of daily living and global cognition. In a more recent study, episodic memory improved in comparison with pretreatment scores in AD patients who received 20 Hz stimulation over the precuneus, whereas no difference was detected after sham stimulation (Koch et al., 2018). A related investigation tested 10 Hz dlPFC stimulation in a mild cognitive impairment (MCI) sample (Drumond Marra et al., 2015) and reported significant benefit relative to sham on tests of everyday memory, with effects persisting up to 1 month. However, in this case, sham group scores for logical memory, executive function, and language also varied over the observation period. Improvements in performance when assessment is repeated over time, or “practice effects,” although controllable with appropriate experimental design, are a frequent confound and complicate interpretation in this area of research. Generally similar results have been reported in other small trials (Eliasova et al., 2014; Rutherford et al., 2015; Zhao et al., 2017), as detailed in Table 1.

Studies directly comparing cognitive outcomes following high-frequency versus low-frequency stimulation in patients with AD were first reported in 2012. In one investigation, 20 or 1 Hz rTMS was delivered bilaterally over the dlPFC in participants with mild or severe dementia (Ahmed et al., 2012). High-frequency stimulation in the mild dementia group was more effective than 1 Hz relative to pretreatment scores as measured by all clinical assessments [i.e., the Mini-Mental State Examination (MMSE), Instrumental Activities of Daily Living Scale (IADL), and the Global Deterioration Scale (GDS)], and the benefits persisted at all test intervals (i.e., up to 3 months). In contrast, participants with severe dementia showed no improvement, regardless of stimulation protocol. A second study also examined the effects of dlPFC stimulation, but in this case, while healthy control subjects received either unilateral iTBS or 1 Hz rTMS, participants with MCI received only unilateral 1 Hz stimulation (Turriziani et al., 2012). Recognition memory improved in both cognitively healthy and MCI subjects following low-frequency stimulation of the right dlPFC compared with sham. Intriguingly, high-frequency TMS over the same site in control subjects impaired recognition memory, raising the possibility that the cognitive response to TMS is dependent on stimulation frequency and/or the baseline status of memory. Unfortunately, the effects of iTBS in memory-impaired participants, with MCI, were not reported (Turriziani et al., 2012).

Encouraged by this background, together with the much larger literature of experimental studies in normal participants (Iriarte and George, 2018), stimulation protocols specifically intended for clinical application in individuals with mild to moderate AD are under active development. Using high-frequency rTMS in conjunction with concurrent cognitive training (rTMS-COG), one current strategy involves an intensive phase of 10 Hz stimulation at six different cortical sites (bilateral dlPFC, parietal somatosensory association cortices, and Broca’s and Wernicke’s areas), nominally three regions/d, 5 d/week for 6 weeks. Alongside rTMS, in this regimen patients receive cognitive training overlapping with TMS delivery, specifically tailored to engage the brain regions targeted for electromagnetic stimulation. A maintenance phase has been included in some studies, composed of two subsequent sessions/week for 3 months. In the first study examining the effects of rTMS-COG (Bentwich et al., 2011), improvement in the AD Assessment Scale-cognitive subscale (ADAS-Cog) was observed at 6 weeks and 4.5 months relative to pretreatment scores. Similar findings in other studies include improved ADAS-Cog and MMSE scores 6 weeks post-treatment (Rabey and Dobronevsky, 2016), and increased ADAS-Cog and Clinical Global Impression of Change (CGIC) scores at 6 weeks, 3 months, and 4.5 months (Rabey et al., 2013) compared with placebo stimulation.

Complementing these findings, rTMS-COG in a group of probable AD case patients reportedly produced statistically significant or numerical improvement relative to baseline, as assessed by a variety of standard measures (e.g., CGIC, MMSE, or ADAS-Cog), either immediately or 6 weeks after the intervention protocol (Lee et al., 2016). Effects were most robust among participants with mild AD and were not detected in those with more advanced cognitive deficits. Notably, however, scores also improved in a parallel sham condition, and, accordingly, interactions between treatment condition and assessment episode were not statistically significant. The endurance of potential treatment benefit in AD remains to be fully documented, but in a recent study (Nguyen et al., 2017) improved ADAS-Cog scores seen 45 d after rTMS-COG reverted to pretreatment baseline at 6 months after intervention. The lack of a sham control that might have detected worse decline without treatment complicates the interpretation of this work (Nguyen et al., 2017).

## Toward an approved TMS therapy for AD

Important issues remain to be addressed in the potential clinical application of rTMS in AD. A recent FDA review for approval of a commercial TMS system for treatment of AD identified a number of deficiencies that need to be addressed, including uncertainty around the reporting of adverse events, concern that current evidence fails to demonstrate a clinically meaningful TMS benefit in AD, and agreement that there are insufficient data documenting that the benefits of the proposed therapy outweigh its health risks (see www.fda.gov, March 21, 2019, Neurological Devices Panel of the Medical Devices Advisory Committee: De Novo DEN160053). The following sections briefly consider some of the experimental design challenges in this area of research, and then turn to evidence concerning the neurobiological mechanisms that might mediate the effects of TMS. A comprehensive mechanistic review is available elsewhere (Keck, 2003; Pell et al., 2011). The perspective here is more targeted, suggesting, on the basis of converging evidence, that NIBS protocols with established safety in healthy young adults may have different effects in the context of AD pathogenesis. Finally, we outline key issues that will need to be resolved in order to advance the rational application of rTMS and related technologies for the prevention, symptomatic relief, or disease-modifying treatment of AD.

## Challenges in assessing rTMS effects in AD

As noted earlier, TMS is approved for the treatment of medication-resistant depression (McClintock et al., 2018; Kaster et al., 2019). Accordingly, in AD trials it is important to control for the possibility that patients might benefit from TMS secondary to stimulation effects on comorbid depressive symptoms (Cotelli et al., 2006, 2008). The prevalence of depression in AD may be as high as 50% (Rutherford et al., 2013), and even mild depressive symptoms are associated with significant functional impairment (Starkstein et al., 2005). Many of the stimulation protocols tested for the treatment of AD are similar to those used in depression, including a prominent focus on the dlPFC. Although some studies have explicitly excluded patients with depression (Cotelli et al., 2006, 2008, 2011; Turriziani et al., 2012; Drumond Marra et al., 2015), others have not reported mood disorders as an exclusion criterion (Ahmed et al., 2012; Rabey et al., 2013; Eliasova et al., 2014; Rabey and Dobronevsky, 2016; Nguyen et al., 2017). The degree to which cognitive improvement following TMS in AD results from alleviating depressive symptoms is therefore difficult to judge, but it is notable in this context that treated patients sometimes also exhibit elevated mood, scoring better on depression and apathy scales (Ahmed et al., 2012; Lee et al., 2016; Nguyen et al., 2017; Padala et al., 2018).

Other experimental controls have also been lacking at times in this area of work. In a number of reports, potential improvements in performance simply as a consequence of repeating cognitive assessment across multiple occasions (i.e., practice effects) were not considered. Studies examining rTMS-COG protocols have generally lacked groups receiving either rTMS alone or cognitive assessment without stimulation (recent preliminary findings are an exception (Alcalá-Lozano et al., 2018), and in such cases the individual and interactive contributions of training and TMS are unknown. Whether they are independent, competitive, or synergistic, there is considerable precedent for the idea that the effects of rTMS are “state dependent” and critically modulated by concurrent functional engagement of the neural circuitry targeted by stimulation (Silvanto et al., 2008). The precise schedule of cognitive training relative to epochs of rTMS delivery, however, has not been systematically manipulated in AD trials. Small sample size is another limitation, and in many investigations, groups of a dozen or fewer participants are not uncommon. Much larger samples, offering increased statistical power, are needed to accurately estimate effect size and enhance reproducibility (Button et al., 2013). As noted in the FDA review cited earlier and in previous reports (Buss et al., 2019; Koch et al., 2019), properly designed, larger, and longer trials are needed to address unresolved issues in the use of rTMS as a therapeutic treatment for AD.

Studies reporting positive TMS effects in mild AD have failed to find reliable benefit in more advanced cases (Ahmed et al., 2012; Rutherford et al., 2015; Lee et al., 2016; Zhao et al., 2017), suggesting that treatment efficacy may be dependent on disease stage. As proposed for other interventions, rTMS might be most effective early in the course of the disease, before neuronal loss has disrupted critical cortical circuitry beyond rescue. The accuracy of early disease diagnosis and staging is an endemic challenge in clinical research on AD, and estimates are that nearly 20% of cases are misdiagnosed (Witte et al., 2014). Thus, important goals for future clinical trials of rTMS include an increased focus on participants qualified on the basis of neuroimaging or biomarker results, and cognitively normal samples at increased risk for the development of disease (e.g., on the basis of APOE genotype or polygenic risk). To date, no longitudinal clinical trial has investigated the response to rTMS-COG in individuals with prodromal or asymptomatic AD, when arresting or reversing neuronal dysfunction may have the greatest prospects of success.

## Potential mechanisms of rTMS benefits

The rTMS protocols tested most frequently as potential interventions for AD were selected partly on the basis of the persistent enhancement in cortical excitability observed following repetitive high-frequency stimulation (Huang et al., 2005; Pötter-Nerger et al., 2009). Such facilitation is thought to involve long-term potentiation (LTP)-like changes in synaptic strength that are widely presumed to be a key cellular mechanism of learning and memory. LTP induced by high-frequency magnetic stimulation (100 Hz) has been directly documented in rat hippocampal slices (Tokay et al., 2009), and related synaptic enhancement has been reported in both other slice preparations and primary cortical cell cultures following 10 and 20 Hz magnetic stimulation (Vlachos et al., 2012; Banerjee et al., 2017). Neuronal activity and LTP regulate the expression of plasticity-related neurotrophins such as brain-derived neurotrophic factor (BDNF), which declines in the AD hippocampus (Phillips et al., 1991), and animal studies confirm that high-frequency rTMS can significantly upregulate BDNF levels (Makowiecki et al., 2014). The speculation based on these findings is that rTMS might result in clinical benefit by correcting or blunting the impaired LTP-like plasticity and associated signaling defects observed in AD (Kumar et al., 2017).

In parallel with these findings, recent advances have also identified rTMS as a modifier of inhibitory neuron function. Studies in hippocampal slice cultures demonstrate that 10 Hz stimulation reduces GABAergic synaptic strength on principal neurons, supporting a model in which mechanisms involving GABAergic synapses modulate overall inhibitory/excitatory balance (Lenz et al., 2016). Findings based on immunocytochemical analysis in animals (Trippe et al., 2009; Mix et al., 2010; Benali et al., 2011) and magnetic resonance spectroscopy in humans (Stagg et al., 2009) show that TMS can lead to temporally graded changes in a variety of inhibitory neuronal markers, lasting at least a week. In preclinical animal research, such alterations generally comprise increases in GABAergic synthesizing enzymes and transporters after low-frequency stimulation (Trippe et al., 2009; Mix et al., 2010; i.e., changes that might promote a net increase in inhibitory drive) and decreases in the number of immunocytochemically identified inhibitory cells after high-frequency stimulation (Benali et al., 2011; Jazmati et al., 2018).

Other mechanisms implicated in the pathogenesis of AD that might contribute to the cognitive effects of rTMS in AD include neurochemical modulation (Michael et al., 2003; Strafella et al., 2003), epigenetic modification of gene transcription (Etiévant et al., 2015), and modulatory effects on neural network dynamics in vulnerable circuitry (Marron et al., 2018). The effect of TMS on these and other potential mechanisms, however, has received limited attention. In the following section, we focus on a particularly illuminating example, suggesting a potential link between the modulatory influence of rTMS on excitatory/inhibitory balance with mechanisms of AD pathogenesis.

## Excitatory/inhibitory balance in AD: a challenging opportunity

Growing interest centers on the possibility that increases in neuronal activity levels directly contribute to AD pathogenesis (Palop et al., 2006). Overexpression of Aβ causes epileptiform activity within entorhinal–hippocampal circuitry that, together with homeostatic responses to aberrant firing, may contribute to memory dysfunction in transgenic mouse models and humans with AD (Palop et al., 2007). Soluble oligomeric Aβ assemblies also increase neuronal excitability and impair hippocampal function by inducing an imbalance between glutamatergic and GABAergic transmission (Lei et al., 2016). The strongest known genetic risk for sporadic AD, the APOE ε4 allele, disrupts GABAergic inhibitory networks, influencing both Aβ aggregation and the clearance of soluble Aβ. In AD mouse models, APOE ε4 knock-in leads to a decrease in GABAergic interneurons in the hilar region of the dentate gyrus that correlates with learning and memory impairment (Li et al., 2009; Huang and Mucke, 2012). This effect, in turn, is reversible with hilar transplantation of inhibitory interneurons (Tong et al., 2014). Relative to noncarriers, the ε4-positive genotype in young adult humans is associated with both hippocampal hyperactivity during memory encoding and increased resting-state connectivity, many decades before clinical or neurophysiological expression of neurodegenerative processes (Filippini et al., 2009). Basic research points to a potential feedforward effect, demonstrating that neuronal stimulation in hippocampal slice preparations induces amyloid precursor protein release (Nitsch et al., 1993), and that stimulating entorhinal cortex projections to the hippocampus increases interstitial Aβ in AD mice (Kamenetz et al., 2003). Thus, together the available findings strongly suggest that neuronal activity is linked to Aβ processing and release, specifically in circuitry known to be affected early in the course of AD (Jagust and Mormino, 2011).

Prompted by the failure of recent clinical trials aimed at slowing or stopping the progression of AD, attention has turned to novel approaches targeting earlier, preclinical abnormalities. Whereas the direction of effect between disrupted neural network activity and AD pathogenesis may vary across brain regions and stages of disease, the emerging consensus is that distributed changes in neuronal excitability are an early signature conferring increased risk for AD (Palop and Mucke, 2010). In this context, therapies aimed at normalizing the balance between excitatory and inhibitory drive in vulnerable circuitry represent a potentially powerful approach to modifying the course of AD. Preliminary support includes evidence that GABA receptor agonist administration in AD transgenic mice (Shao et al., 2014) and aged mice (Yamamoto et al., 2015), as well as in humans (Chung et al., 2016), lowers Aβ burden and attenuates Aβ-induced neurotoxicity. Other treatments, including the use of growth hormone-releasing hormone in healthy elderly and MCI subjects, increase cortical GABA levels in association with improved cognition (Friedman et al., 2013). In perhaps the most direct test of targeting excess neuronal activity, low-dose treatment with the antiepileptic levetiracetam improves memory in both aged rats (Koh et al., 2010) and individuals with amnestic MCI (Bakker et al., 2012), together with a reduction in hippocampal hyperactivity. Whether this approach, implemented early, is sufficient to alter the fundamental trajectory of disease is under active investigation.

## Frontiers in AD management and treatment using TMS: a path forward

The possibility that a safe, noninvasive, and relatively low-cost treatment such as TMS might prove effective in the battle against AD has generated understandable excitement (https://www.scientificamerican.com/article/could-magnetic-brain-stimulation-help-people-with-alzheimer-rsquo-s/). However, the available evidence regarding clinical efficacy and mechanism of action is limited. The view developed here is that defining the neurobiological substrates responsible for the effects of TMS and other NIBS modalities will be critical for maximizing their efficacy and safety. We encourage a constructive, dispassionate evaluation of the evidence, aimed at establishing an informed platform for moving TMS and related strategies forward toward clinical application in AD.

**Figure 1.**
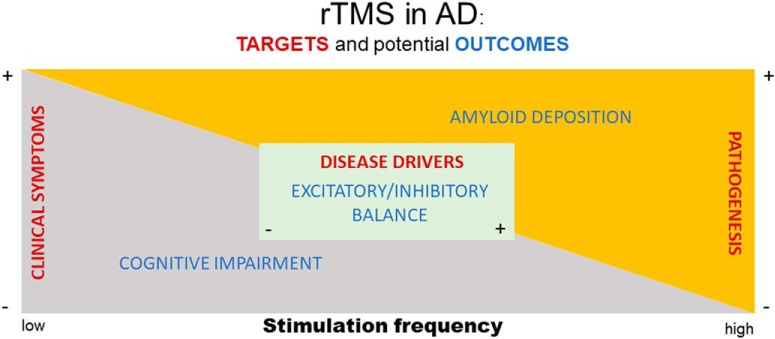
Schematic representation of target areas (red text) and potential outcome measures (blue text) to test rTMS as an intervention for AD. See text for further description.

The evidence summarized in this review highlights at least three conceptually distinct targets for TMS intervention in AD. Figure 1 schematically represents the hypothetical relationships between these targets (red text), together with an exemplar outcome for each (blue text), and how they might vary with low- versus high-frequency TMS. The majority of extant research in this area has examined stimulation effects on cognitive and neuropsychological symptoms of disease, with the primary outcome of interest comprising improved clinical outcome. The effects of high-frequency stimulation have been tested most often, with positive studies reporting a variable degree of cognitive benefit, at least in mild AD. Insufficient attention has been directed at tracking the influence of TMS on AD pathogenesis or biomarkers (i.e., proxies of the underlying disease process; for example, see Marron et al., 2018). Nonetheless, substantial evidence indicates that neuronal activity promotes amyloid deposition, raising the possibility that the same high-frequency stimulation that leads to improved clinical symptoms might also accelerate underlying AD pathogenesis. Conversely, low-frequency rTMS reportedly decreases amyloid burden in the brains of AD transgenic mice (Huang et al., 2017), while preserving the reported cognitive benefit of high-frequency stimulation. Finally, perhaps the most hopeful target of TMS in AD—that intervening before symptom onset might correct contributing mechanisms or block seed events in the initiation of the disease process—remains largely untested. Disrupted excitatory/inhibitory balance is thought to comprise an early driver of AD pathogenesis, and, based on its presumed mechanism of action, TMS may be ideally positioned as a disease-modifying intervention against this target.

The need for effective strategies in the battle against AD grows ever more urgent. The disappointing outcome of recent clinical trials encourages the consideration of fresh perspectives, and in this context, NIBS has emerged as a novel alternative to pharmacological therapeutics and other interventions. The exciting potential of this approach, however, should not overshadow the important questions that remain unanswered. Among them, the safety profile established for other indications merits reconsideration in the context of neurobiological changes associated with AD, including hyperexcitability and epileptic activity, consistent with current safety guidelines (Rossi et al., 2009). Studies aimed at directly tracking pathological progression by *in vivo* imaging in patients receiving TMS are also needed. Efficacy in appropriately controlled, well powered trials remains to be confirmed, and longer-term cognitive outcomes established. At what stage in the progression of AD pathology will TMS be most effective? If TMS is used to target excitatory/inhibitory balance, at what frequency and in which brain regions, recognizing that such effects may be brain region specific (Bañuelos et al., 2014)? Indeed, given the prominent regional vulnerability of AD, it will be important to consider that TMS aimed at correcting excitatory/inhibitory balance in one target area may well have unanticipated or negative secondary effects in other, distally connected networks. Basic research, designed in alignment with the priorities of clinical research, can provide helpful guidance and yield much needed insight into the neurobiological mechanisms responsible for the clinical effects of NIBS (Tang et al., 2017). The challenges are great, but a path forward toward the rational application of rTMS and related modalities in AD has begun to emerge.

## Synthesis

Reviewing Editor: Jorge J. Palop, Gladstone Institutes and UCSF Decisions are customarily a result of the Reviewing Editor and the peer reviewers coming together and discussing their recommendations until a consensus is reached. When revisions are invited, a fact-based synthesis statement explaining their decision and outlining what is needed to prepare a revision will be listed below.

This is overall a thoughtful and interesting review describing an emerging field with therapeutic potential in AD. As described below, the review needs to be more specific for many statements describing conditions and therapeutic benefits. Additional subheadings and illustrations will be of great value for clarifying and organizing the content of the review.

Specific comments:

Abstract, “The mechanistic basis of therapeutic benefit is poorly understood but is thought to involve the enhancement of synaptic efficacy in memory-related neural networks.” Move this sentence after the description of therapeutic benefits of TMS.

Abstract, “on the possibility that treatment might influence excitatory/inhibitory balance.” I will change “treatment” to “TMS stimulation”. I will avoid the use of the term “treatment” since “TMS stimulations” are currently not treatment or standard care for AD people.

Abstract, “we emphasize that TMS treatments established in young adults may have unanticipated effects in older individuals or in the brain compromised by AD pathology.” Again, clarify this sentence, “treatments”, “young adults”, “unanticipated”. Young adults with AD or healthy young adults? If healthy, is this a treatment to provide care? Describe the “Unanticipated effects”. Does “unanticipated” mean “detrimental effects on cognition”?

The significant statement describes much better and with precision the current status and challenges for TMS, including the final sentence. This type of language should be included in the abstract.

Page 3, “The single greatest risk for AD is aging. The ε4 allele of the apolipoprotein E (APOE) gene is present in approximately 40% of cases and is the strongest genetic risk for the sporadic, late-onset form of AD (Farrer et al., 1997)”. Please verify this number with other publications, I believe the % of AD cases with APOE4 is around 60-80%.

Page 4, “well-tolerated, low risk safety profile in healthy adult controls, with established efficacy for a growing rage of conditions”. It would be useful to describe the standard regimens for TMS and tDCS (eg., repetitive, acute, frequency, daily, weekly, monthly), the conditions/indications and efficacy. These introductory statements need more precession to be meaningful for the reader. I think it is important to be specific describing the benefits and the conditions.

Page 4, “Among NIBS modalities, TMS has enjoyed the widest application in clinical research

on neuropsychiatric disorders.” Again, please be specify about the disorders and the symptoms before discussing the potential mechanisms. Widest application?

Page 4, Adding an illustration about the different stimulation protocols and effects on cortical suppression/inhibition or facilitation/excitation will be very useful, particularly if indications and symptoms can be included. This could be a subheading, which may include the first paragraph of “1. Testing TMS as an Intervention for AD”, which also describes stimulation protocols.

Page 4, the original descriptions of high and low frequencies stimulations are <1 or >3 Hz, but latter only 20 Hz stimulations are introduced as high. Please include ranks if >3Hz also includes 20 Hz (e.g., >3-20Hz) or maybe describe also 20Hz.

It would help the readers to add more subheadings to make the structure more evident. Subheading could be more informative providing a conclusion (e.g., “testing TMS as intervention for AD” to “Therapeutics effects of TMS in AD patients” if this is the overall conclusion).

Page 4, “1. Testing TMS as an Intervention for AD” should be “2.”

Page 8, add a subheading starting with the FDA paragraph seems appropriate. These concepts need to be expanded.

Page 9, I would describe first the conditions or symptoms that TMS clearly produce benefits, for example depression before introducing “the testing in AD patients”. I think it is important start with clear clinical data that this approach works for some disorders to educate the reader and move to Alzheimer.

Page 9, the concept of “practice effects” for repeated testing is not clear, testing is used as technique “testing TMS” but here refers to “cognitive testing”.

Pages 9 and 10, The concept of “cognitive training” as an experimental group is not clear. I think it means “cognitive testing without stimulation” but not specific cognitive training. Is the training related to the repeated cognitive testing?

Page 11, a diagram of potential mechanisms would be useful.

Page 11, “Other mechanisms that might contribute to the cognitive effects of rTMS in AD

include neurochemical modulation (Michael et al., 2003; Strafella et al., 2003), epigenetic

modification of gene transcription (Etievant et al., 2015), and modulatory effects on neural

network dynamics in vulnerable circuitry (Marron et al., 2018).” Please be more specific about “neurochemical modulation”, “epigenetic modification of gene transcription” and “network dynamics”. These sentences need to be expanded to provide meaningful insights. These concepts could be expanded since it is the topic of the review.

These two subheadings are overlapping, since excitatory/inhibitory balance is a proposed mechanism.

3. TMS: Potential Mechanisms of Action

4. Excitatory/Inhibitory Balance in AD: A Challenging Opportunity

Maybe 3. should be “synaptic plasticity, neuromodulation, and neurotrophic effects”.

Page 11, “Studies in hippocampal slice cultures demonstrate that 10 Hz stimulation reduces

GABAergic synaptic strength on principal neurons”, indicate that 10 Hz is electromagnetic stimulation.

Page 12, “4. Excitatory/Inhibitory Balance in AD: A Challenging Opportunity”. Consider to divide this section in mechanisms of TMS stimulation on GABA (combined with the previous section about mechanism) and AD pathogenesis and challenging opportunity.
